# Fusion of the *Dhfr*/Mtx and IR/MAR Gene Amplification Methods Produces a Rapid and Efficient Method for Stable Recombinant Protein Production

**DOI:** 10.1371/journal.pone.0052990

**Published:** 2012-12-31

**Authors:** Chiemi Noguchi, Yoshio Araki, Daisuke Miki, Noriaki Shimizu

**Affiliations:** 1 Graduate School of Biosphere Science, Hiroshima University, Higashi-hiroshima, Hiroshima, Japan; 2 Tosoh Co., Ayase, Kanagawa, Japan; Naval Research Laboratory, United States of America

## Abstract

Amplification of the dihydrofolate reductase gene (*Dhfr*) by methotrexate (Mtx) exposure is commonly used for recombinant protein expression in Chinese hamster ovary (CHO) cells. However, this method is both time- and labor-intensive, and the high-producing cells that are generated are frequently unstable in culture. Another gene amplification method is based on using a plasmid bearing a mammalian replication initiation region (IR) and a matrix attachment region (MAR), which result in the spontaneous initiation of gene amplification in transfected cells. The IR/MAR and *Dhfr*/Mtx methods of gene amplification are based on entirely different principles. In this study, we combine these two methods to yield a novel method, termed the IR/MAR-*Dhfr* fusion method, which was used to express three proteins, the Fc receptor, GFP, and recombinant antibody. The fusion method resulted in a dramatic increase in expression of all three proteins in two CHO sub-lines, DXB-11, and DG44. The IR/MAR-*Dhfr* fusion amplified the genes rapidly and efficiently, and produced larger amounts of antibody than the *Dhfr*/Mtx or IR/MAR methods alone. While the amplified structure produced by the *Dhfr*/Mtx method was highly unstable, and the antibody production rate rapidly decreased with the culture time of the cells, the IR/MAR-*Dhfr* fusion method resulted in stable amplification and generated clonal cells that produced large amounts of antibody protein over a long period of time. In summary, the novel IR/MAR-*Dhfr* fusion method enables isolation of stable cells that produce larger amounts of a target recombinant protein more rapidly and easily than either the *Dhfr*/Mtx or IR/MAR methods alone.

## Introduction

Amplification of oncogenes or drug-resistance genes plays a pivotal role in malignant transformation of human cells via over-production of the corresponding protein products; therefore, understanding the gene amplification process has important implications for cancer research (reviewed in [1]–[3]). Because the frequency of spontaneous gene amplification in mammalian cells is extremely low, a method that can be used to mimic gene amplification in cultured cells is required. Several cytotoxic drugs induce amplification of genes that confer resistance to the individual drugs; the first study in this field was performed half a century ago [4] and has been reviewed more recently [5]. The most widely used cytotoxic drug-induced gene amplification method involves amplification of the dihydrofolate reductase gene (*Dhfr*), alone or together with physically linked genes, in Chinese hamster ovary (CHO) cells that lack a functional *Dhfr* gene, under the selection pressure of increasing concentrations of methotrexate (Mtx). Mtx is an inhibitor of DHFR, which catalyzes nucleotide synthesis; therefore, DNA replication is arrested under conditions where DHFR is completely inhibited. The *Dhfr*/Mtx method has been used extensively in investigations of the mechanism of gene amplification (reviewed in [6]) and has been the most commonly used approach in industrial recombinant protein production (reviewed in [7]).

Because the production of protein pharmaceuticals, including cytokines and humanized antibodies, depends on the use of cultured mammalian cells, a robust method for the production of large amounts of recombinant protein in mammalian cells has become increasingly important (reviewed in [7]). Although the *Dhfr*/Mtx method has been successfully used to obtain high-producer clones, it is particularly time and labor-intensive and obtaining high-producer clones requires repeated cycles of selection and cloning [8]. Furthermore, high-producer clones obtained by the *Dhfr*/Mtx method are frequently unstable [9] and show a rapid decrease in protein synthesis as cell culture time progresses; this form of instability was also reported for a method that amplifies the glutamine synthetase gene under induction by methionine sulfoximine [10]–[12].

We previously found that plasmids bearing a mammalian replication initiation region (IR) and a nuclear matrix attachment region (MAR) are spontaneously amplified in transfected cells, and efficiently generate chromosomal homogeneously staining regions (HSRs) and/or extrachromosomal double minutes (DMs) [13], [14], which are cytogenetic manifestations of gene amplification. IR/MAR-induced gene amplification is highly efficient, is detected in almost all transfectants, and is spontaneous, requiring selection based only on standard drug resistance to blasticidin or neomycin, for example. Efficient gene amplification is not supported by either IR or MAR alone, or by an unrelated sequence of equal size [13], [15]. We previously proposed a model to explain how the IR/MAR plasmid spontaneously undergoes amplification in transfected cells, in which the plasmid is initially amplified to a large circular extrachromosomal molecule containing multiple copies of the original plasmid sequence arranged as direct repeats [14]. This extrachromosomal amplification occurs spontaneously and requires the IR/MAR sequence ([14], [15] and our unpublished data); if the extrachromosomal amplification step proceeds for an extended magnitude, the amplified DNA appears cytogenetically as DMs. Damaged extrachromosomal circular DNA is eliminated from the cells through the generation of micronuclei [16]. Alternatively, if the extrachromosomal molecule is integrated in the arm of the chromosome, the plasmid repeat spontaneously initiates the breakage-fusion-bridge (BFB) cycle, which generates a long HSR composed of a homogeneous array of plasmid sequences in human colorectal carcinoma COLO 320DM cells [14], [17]. Because the HSR is generated by the BFB cycle, it contains many inverted recombined sequences [18]. By contrast, in CHO cells the plasmid repeat rarely initiates the BFB cycle, and most of the repeat remains unamplified at the chromosome arm ([19] and our unpublished data). Alternatively, the BFB cycle may be initiated in these cells at low frequency in the sequence flanking the repeat; consequently, this process generates a fine ladder-type HSR that differs in appearance from the homogeneous HSR seen in COLO 320 cells. The IR/MAR gene amplification system has been used in basic cell biology research (reviewed in [20]), and has been adapted for recombinant protein production. This method has been shown to dramatically improve production of green fluorescent protein (GFP) in human COLO 320 cells [21], human cyclooxygenase-1 in human HEK 293T cells [22], and humanized antibody in CHO DG44 cells [19].

Because IR/MAR and *Dhfr*/Mtx amplification are based on different mechanisms, we hypothesized that the two methods might interact synergistically to provide a more valuable method for recombinant protein production. Here, we describe such a combined approach, referred to as the IR/MAR-*Dhfr* fusion or IR/MAR-fusion method. We found that Mtx treatment extended the chromosomal distribution of the *Dhfr*-bearing IR/MAR plasmid and effectively generated a fine ladder HSR in CHO cells. This novel fusion approach yields more stable transfectants that produce larger amounts of recombinant protein more quickly and efficiently than either the IR/MAR or *Dhfr*/Mtx methods alone.

## Materials and Methods

### Construction of Plasmids

**Figure 1:**
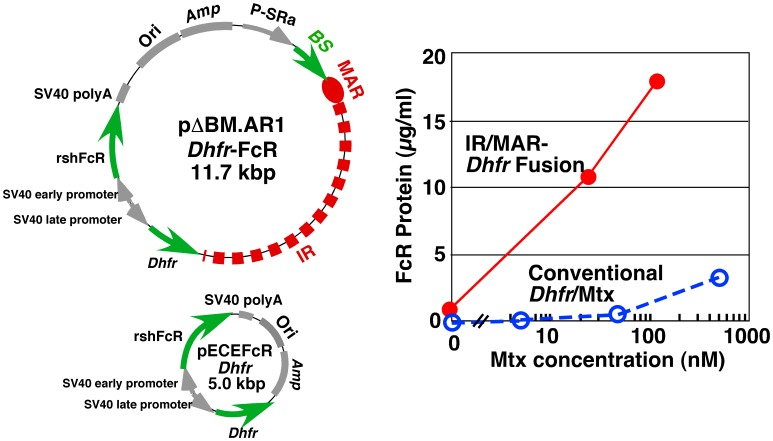
*FcR* plasmids and expression of the *FcR* gene. CHO DXB-11 cells were transfected with the IR/MAR-positive pΔBM AR1*Dhfr*-FcR plasmid or the IR/MAR-negative pECE-FcR *Dhfr* plasmid [23_ENREF_23] by electroporation, and then cultured for 10 days in α-MEM(−). Clones were obtained by limiting dilution; the highest producer of FcR was determined by ELISA and then cultured in the presence of 25 nM Mtx for 15 days. Surviving cells were subjected to another round of limiting dilution and the highest producers were then cultured in the presence of 125 nM Mtx for 20 days. The same selection process was used for clones obtained using the conventional *Dhfr*/Mtx method; however, cells were selected and cloned in medium containing 5, 50 and 500 nM Mtx. After each round of selection, the level of FcR protein (µg/ml) was determined by ELISA.

**Figure 2:**
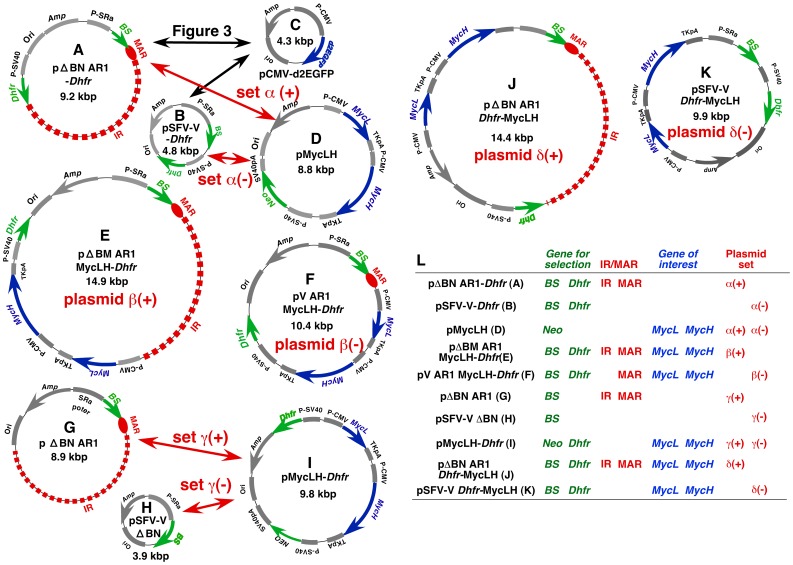
Plasmid structures. In panels A to K, plasmid sets α to δ are labeled in red; (+) and (−) indicate the presence and absence of IR/MAR, respectively. Double-headed red arrows indicate the co-transfected plasmid set α and γ. Double-headed black arrows indicate the two sets of co-transfected plasmids used to quantify *GFP* expression by flow cytometry (Figure 3). In each plasmid, blue arrows represent the target genes (*d2EGFP*, *MycL,* and *MycH*) and green arrows represent the drug-resistance genes (*Dhfr*, *BS,* and *Neo*); the IR and MAR are represented by a red dashed bold line and red oval, respectively. The promoters (P-CMV, P-SRα or P-SV40), polyA-addition sequences (TK-pA or SV40-pA), and sequences required for cloning in *E. coli* (Ori and Amp), are shown in gray. In panel L, the features of the plasmids are summarized. The font colors are the same as those used in panels A to K.

The pΔBM AR1 *Dhfr*-FcR plasmid (Figure 1) was constructed by inserting the *Bam*HI fragment of pECE-rshFcR *Dhfr* [23], which contains the *Dhfr* gene and the recombinant soluble human Fc receptor (*rshFcR*) gene expression cassettes, in place of the *hyg* expression cassettes (*Bam*HI-*Nru*I) in pΔB AR1 [14]. pΔB AR1 contains an IR sequence from the 3′-noncoding region of the hamster *Dhfr* locus and a MAR sequence (AR1) from the immunoglobulin (*Ig*) *κ* intron. pCMV-d2EGFP (Figure 2C) was previously described [21] and contains the plasmid backbone and the *d2EGFP* expression cassette, which encodes a GFP derivative with a short intracellular half-life. pΔBN AR1 (Figure 2G) was previously described [14] and contains an IR and a MAR sequence, as in pΔB AR1. Plasmids expressing either the light (pMyc L) or heavy (pMyc H) chain of humanized anti-c-MYC (9E10) antibody, or both (pMyc LH; Figure 2), were recently described [19].

Four additional plasmid sets (α to δ; Figure 2) were used to precisely evaluate the synergistic action of IR/MAR-mediated and *Dhfr*/Mtx-mediated gene amplification. To construct plasmid set α, the SV40 promoter from pMycL and the *Dhfr* protein-coding sequence from pOptiVEC-TOPO (Invitrogen) were amplified by PCR and the products were ligated in that order. The resulting DNA fragment was inserted either in place of the *Hyg* expression cassette (*Bam*HI/*Nru*I fragment) of pSFV-V [13], or at the *Bam*HI site of pΔBN AR1, using the In-Fusion® HD Cloning Kit with Cloning Enhancer (Invitrogen), to generate pSFV-V-*Dhfr* (Figure 2B) or pΔBN AR1-*Dhfr* (Figure 2A), respectively. These plasmids differ only by the presence of the IR/MAR sequence in pΔBN AR1-*Dhfr*. For co-transfection, pSFV-V-*Dhfr* and pMycLH are referred to as plasmid set α(−) and pΔBN AR1-*Dhfr* and pMycLH are referred to as plasmid set α(+). It has previously been shown that any plasmid co-transfected with the IR/MAR plasmid is co-amplified [14].

To construct plasmid set ß, the SV40 promoter and the downstream *Dhfr* gene were amplified from pΔBN AR1-*Dhfr* (of plasmid set α(+)) and inserted at the *Mlu*I site of pBM-MycLH as previously described [19]. The resulting plasmid, pΔBM AR1 MycLH-*Dhfr* (Figure 2E), contained the IR/MAR region and was designated plasmid ß(+). The IR sequence was removed from pΔBM AR1 MycLH-*Dhfr* by digestion with *Mfe*I/*Kpn*I, blunted and self-ligated. The resulting plasmid, pV AR1 MycLH-*Dhfr* (Figure 2F), contained the MAR (AR1) but not the IR sequence and was designated plasmid ß(−).

The DNA fragment bearing the SV40 promoter and *Dhfr* gene used to construct plasmid set β was also used to construct plasmid set γ. This fragment was inserted in the *Mfe*I site, located between the CMV promoter and the *Amp* gene, to generate pMycLH-*Dhfr* (Figure 2I). pMycLH-*Dhfr* was co-transfected with pΔBN AR1 or pSFV-V ΔBN as plasmid set γ(+) or γ(−), respectively. As described for plasmid set β these sets differ only by the presence of the IR/MAR in plasmid set γ(+).

To construct plasmid set δ, a DNA fragment containing the *Ig* light and heavy chain gene expression cassette was isolated from pMycLH by digestion with *Acl*I and *Nae*I, which cut at the 5′-flanking region of the CMV promoter driving the *MycL* gene and the 3′-flanking region of the TK poly A sequence that terminates the *MycH* gene, respectively. The DNA fragment was inserted at the *Aat*II site of either pΔBN AR1-*Dhfr* or pSFV-V-*Dhfr*, to produce pΔBN AR1 *Dhfr*-MycLH (plasmid δ(+); Figure 2J) or pSFV-V-*Dhfr*-MycLH (plasmid δ(−); Figure 2K), respectively.

### Cell Culture, Transfection, Selection, and Cloning

The *Dhfr-*deficient hamster cell lines, CHO DXB-11 and CHO DG44, were obtained from Dr. Lawrence Chasin of Columbia University. Cells were grown in Minimum Essential Medium with nucleosides and deoxynucleosides (Sigma) (αMEM(+)), supplemented with 10% fetal bovine serum (FBS). For passage, cells were dissociated using TrypLE Express® with Phenol Red (Invitrogen). Cultures were periodically tested for the presence of mycoplasma.

In the experiment shown in Figure 1, CHO DXB-11 cells were transfected with IR/MAR-positive pΔBM AR-*Dhfr*-FcR (Fig. 1A) or IR/MAR-negative pECE-rshFcR *Dhfr* [23] by electroporation using the GenePulser Xcell (Bio-Rad), and were cultured for 10 days in α-MEM without nucleosides and deoxynucleosides (α-MEM(-)). Clones were obtained by limiting dilution; the highest producer of FcR was determined by ELISA and was cultured in the presence of 25 nM Mtx for 15 days. Surviving cells were subjected to another round of limiting dilution and the highest producers were then cultured in the presence of 125 nM Mtx.

**Figure 3:**
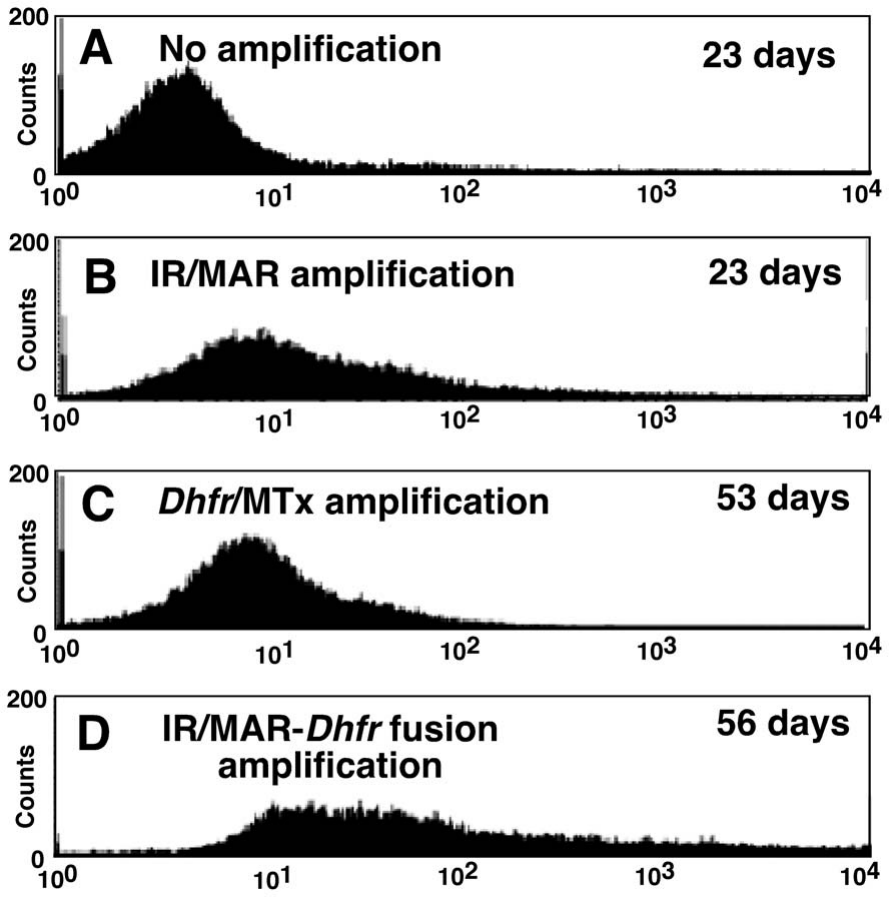
Quantitation of *d2GFP* gene expression by flow cytometry. CHO DG44 cells were transfected with the pCMV-d2EGFP plasmid and IR/MAR-negative pSFV-V-*Dhfr* (A, C) or IR/MAR-positive pΔBN AR1-*Dhfr* (B, D) plasmid. These vector sets are indicated by the double-headed black arrows in Figure 2. Cells were then selected with 10 µg/ml blasticidin in αMEM(+)(A and B), or with 10 µg/ml blasticidin and 5 nM Mtx in αMEM(−) (C and D). Stable transfectants produced without gene amplification (A), with IR/MAR-amplification (B), *Dhfr*/Mtx amplification (C), or IR/MAR-*Dhfr* fusion amplification (D), were obtained and d2GFP fluorescence was detected using flow cytometry. The number of culture days at the time of analysis is noted in each panel.

In the experiment shown in Figure 3, CHO DG44 cells were co-transfected with the IR/MAR-negative plasmid combination (pCMV-d2EGFP and pSFV-V-*Dhfr*; Figure 2B and C), or the IR/MAR-positive plasmid combination (pCMV-d2EGFP and pΔBN AR1-*Dhfr*; Figure 2A and C). Transfectants from the IR/MAR-negative and IR/MAR-positive plasmid pairs were selected with 10 µM blasticidin in α-MEM(+) (Figure 3A, “No amplification” and Fig. 3B, “IR/MAR-amplification”, respectively), or 10 µM blasticidin followed by 5 nM methotrexate in α-MEM(−) (Figure 3C, “*Dhfr*/Mtx amplification” and Figure 3D, “IR/MAR-*Dhfr* fusion amplification”, respectively). Flow cytometric analysis of d2EGFP expression was performed using the FACSCalibur instrument (Becton Dickinson).

All other transfections were performed in CHO DG44 cells by lipofection with Lipofectamine 2000 (Invitrogen), according to the manufacturer’s recommended protocol. Twenty-four to 30 hours after transfection, cells were replated onto 10 cm dishes in fresh α-MEM(−), containing 10% dialyzed FBS (Hyclone), in the presence or absence of 10 µg/ml blasticidin (Kaken Pharmaceuticals) or 500 µg/ml Geneticin 418 (Sigma). Hundreds to several thousand colonies, approximately 0.5 mm in diameter, usually appeared 11 to 13 days after transfection; at this stage cells were replated in the selection media described above, supplemented with 5 nM Mtx (Sigma). The small proportion of surviving, 5 nM Mtx-adapted cells reached confluence in 10 cm dishes after 23 to 32 days. Ten percent of these cells were then replated in 10 cm dishes and cultured in medium containing 50 nM Mtx. The remaining 5 nM Mtx-adapted cells were analyzed as described below. Increasing the Mtx concentration from 50 nM to 500 nM was acheived in the same manner. All experiments were performed using adherent CHO DG44 or DXB-11 cells.

### Fluorescence in situ Hybridization

Fluorescence in situ hybridization (FISH) analysis of metaphase chromosome spreads was performed using a DIG-labeled probe prepared from pΔBN.AR1 (Figure 2A) plasmid DNA, which hybridizes to the plasmid backbone sequence and the IR/MAR, as previously described [14]. We previously demonstrated that co-transfected plasmids are ligated in the cells shortly after transfection and co-amplified; therefore, the alternation of the co-transfected plasmids could be observed in the chromatin fiber, while both signals were completely intermixed in the metaphase chromosome, which has an extremely compact structure [14].

### ELISA

Cells were seeded in 96-well plates at a density of 1×10^5^ cells/0.2 ml in the absence of a selective drug, and in the presence or absence of 10 mM sodium butyrate (Sigma). After 3 days of culture, the medium was harvested and analyzed by sandwich ELISA using rabbit anti-human IgG (The Jackson Laboratory) as the immobilization antibody, and purified human IgG (ZYMED) as the standard preparation. Horseradish peroxidase-conjugated anti-6-His antibody (Bethyl Laboratories, Inc.) was used for the quantification of recombinant FcR.

### Real-time PCR

Cells were harvested and genomic DNA was extracted by standard methods using SDS and proteinase K. Real-time PCR was performed on a StepOnePlus™ system (Applied Biosystems) with Thunderbird™ qPCR Mix (Toyobo) and gene-specific primers for *β-actin*, *MycL*, and IR/MAR. The relative copy numbers of *MycL* and IR/MAR were normalized to that of *β-actin*. Primer sequences and PCR conditions can be provided upon request.

## Results and Discussion

### Expression of the Fc Receptor

We compared amplification of Fc receptor gene (*FcR*) expression using the IR/MAR-*Dhfr* fusion and conventional *Dhfr/*Mtx methods. CHO DXB11 cells were transfected with the IR/MAR-positive pΔBM AR1*Dhfr*-FcR plasmid (Figure 1A) or the IR/MAR-negative pECE-rshFcR *Dhfr* plasmid [23]. The clone showing the highest level of FcR production obtained by each method was then cultured in increasing concentrations of Mtx. Compared with the cultures produced by the conventional *Dhfr/*Mtx method, *FcR* expression increased much more rapidly in the IR/MAR-*Dhfr* fusion cultures (Figure 1); detection of the plasmid sequence by FISH suggested that this amplification progressed rapidly and dramatically (data not shown). It was previously reported that the IR/MAR plasmid is not efficiently amplified in CHO-K1 cells and that only a small amount of recombinant protein is expressed [19]. Similar results were obtained with CHO DXB11 cells (our unpublished data). While CHO-K1 and CHO DXB11 are of the same lineage, CHO DG44 is of a different lineage [24]. However, our results demonstrate that, if used in combination with *Dhfr*/Mtx amplification, the IR/MAR sequence enables rapid acquisition of high-producer cells even in CHO DXB11 cells (Figure 1B), and suggests synergism between the two gene amplification methods.

### Expression of d2EGFP

We then compared the IR/MAR-*Dhfr* fusion method to the IR/MAR and *Dhfr*/Mtx methods for expression of the *d2EGFP* gene in CHO DG44 cells. Flow cytometric analysis of the polyclonal transfectants revealed that, at the same time after transfection (23 days), the original IR/MAR method produced a cell population that expressed significantly higher levels of GFP (Figure 3B) than the selection method using the control, non-amplifiable vector (Figure 3A). The conventional *Dhfr*/Mtx method (Figure 3C) produced cells expressing a level of GFP that was higher than the control non-amplifiable method (Figure 3A), but less than or equal to the IR/MAR method (Figure 3B). Compared with the other methods, the IR/MAR-*Dhfr* fusion method generated a significant proportion of cells that expressed a very high level of GFP (Figure 3D) and the time required until the analysis was similar for the *Dhfr*/Mtx and IR/MAR-*Dhfr* fusion methods (53 and 56 days, respectively). These data suggest that the IR/MAR-*Dhfr* fusion method has strong merit over the original IR/MAR method or the conventional *Dhfr*/Mtx method for acquisition of cells that produce larger amounts of recombinant protein.

### Amplification and Expression of Antibody Using Plasmid Set α

We next examined the expression of an industrially important protein, recombinant antibody, in CHO DG44 cells. Plasmid sets α(−) and α(+) (Figure 2A, B, and D), consisting of pMycLH, which contains the expression cassettes for the heavy (*MycH*) and light (*MycL*) chain genes of humanized anti-c-*myc* antibody, and either pΔBN AR1-*Dhfr* (IR/MAR-positive) or pSFV-V-*Dhfr* (IR/MAR-negative), which were used for the expression of *d2EGFP*, were employed. Polyclonal transfectants were selected without cloning, by culture in nucleotide-deficient medium containing blasticidin and increasing concentrations of Mtx.

**Figure 4:**
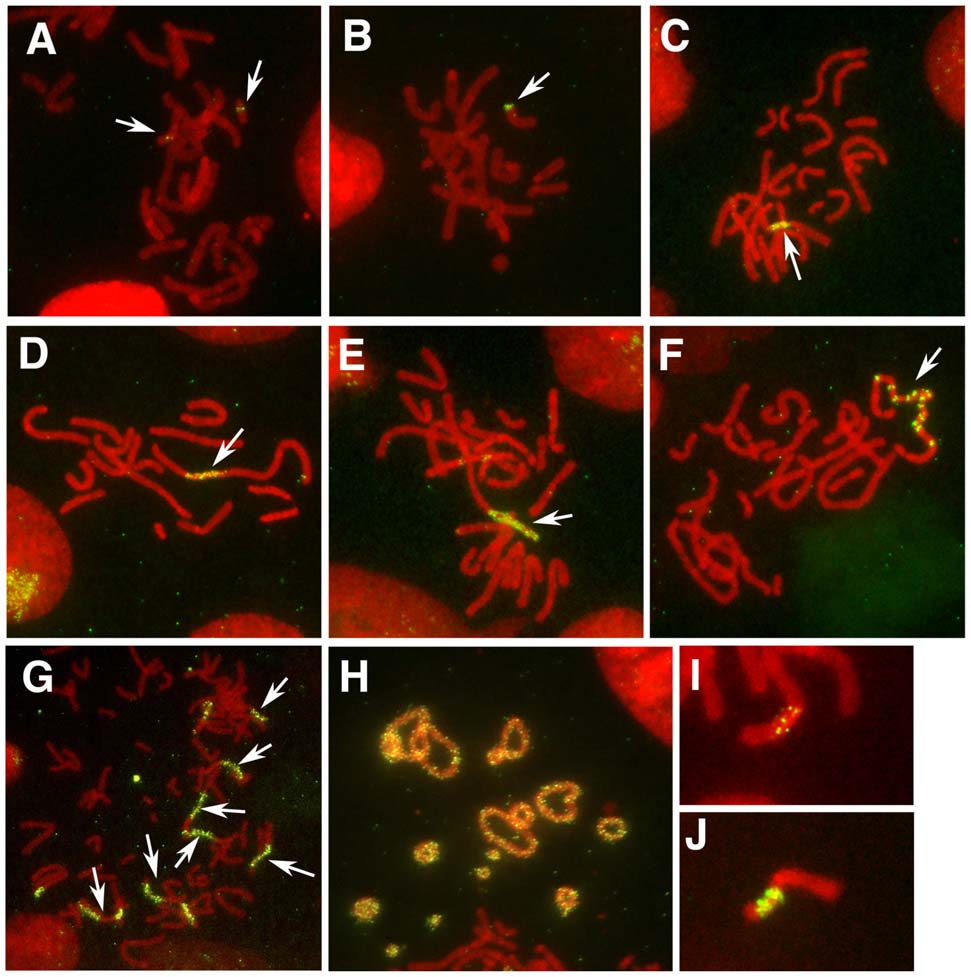
Cytogenetic appearance of amplified sequences. FISH analysis of metaphase spreads prepared from transfected CHO DG44 cells showing dots (A); lines (B); a fine ladder HSR of approximately two (C, I, and J), four (D), or six (E) chromosome widths (cw); and a ladder HSR (F). The frequencies of these structures were determined and are shown in Figures 5 to 7. Multiple HSRs in a single cell (G) or ring chromosomes consisting of ladder HSRs (H) were observed in transfectants grown in 500 nM Mtx. The densities of the plasmid signals in the HSR shown in panels I and J are clearly different; however, both were classified as two cw, because the signals are distributed along the length of two cws.

**Figure 5:**
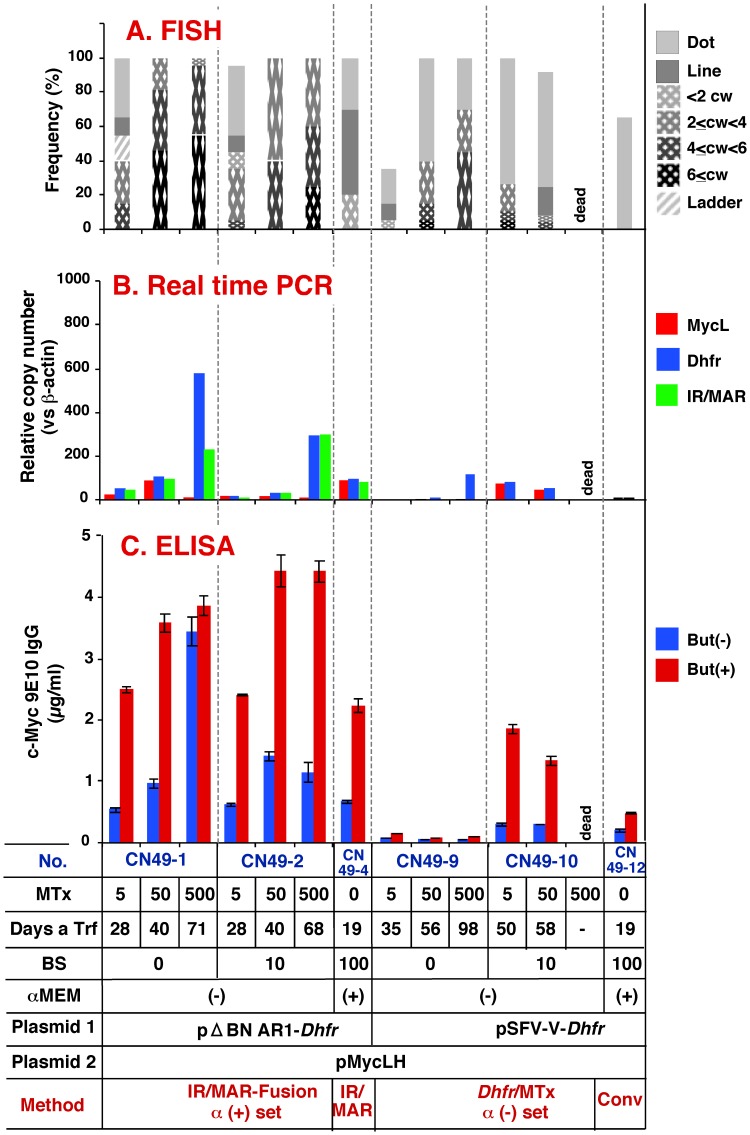
Amplification and antibody expression using plasmid set α**.** CHO DG44 cells were co-transfected with pMycLH (plasmid 2) and pΔBN AR1-*Dhfr* or pSFV-V-*Dhfr* (plasmid 1); cells were then selected by culture in the presence of 500 µg/ml G418, and the indicated concentrations of blasticidin (BS) and Mtx. The transfectant number (No.), Mtx concentration (nM), BS concentration (µg/ml), αMEM used (with (αMEM +) or without (αMEM−) nucleosides and deoxynucleosides), and amplification method, are indicated at the bottom of the figure. IR/MAR: IR/MAR method; Conv: conventional expression plasmid. Cells reached confluence at the indicated number of days after transfection (Days a Trf). Cytogenetic structures were analyzed by FISH (A) (cw: chromosome width). Antibody expression was quantified by real-time PCR (B), and ELISA (C). Cells prepared for ELISA were grown in the presence (+) or absence (−) of 10 mM sodium butyrate (But). Error bars represent mean +/− S.D.

FISH analysis revealed that the plasmids generated varying lengths of fine ladder-type HSRs, consisting of arrays of tiny dots along the chromosome arm (Figure 4A to E), or ladder-type HSRs, where the plasmid sequences were separated by segments of unlabeled chromosomal material (Figure 4F); this result was consistent with our previous report [19]. The length of the HSRs was recorded and the frequency was plotted (Figure 5A). Fine ladder-type HSRs were also observed, in which the plasmid signals were either sparsely (Figure 4I) or densely (Figure 4J) arrayed. The signal size also appeared to vary among cells. The appearance of these signals is related to the mechanism of amplification; therefore, these observations merit further investigation. The length of the HSRs detected by FISH did not necessarily reflect the copy number of the genes; therefore, PCR analysis was carried out in parallel. The PCR data revealed that the IR/MAR-negative plasmid grown in nucleotide-containing medium (pSFV-V-*Dhfr*; conventional expression vector; transfectant no. CN49-12) generated only a low level of amplified structure, whereas the IR/MAR-positive plasmid (pΔBN AR1-*Dhfr*; IR/MAR method; CN49-4) generated a longer HSR at a higher frequency under the same conditions after one-step selection in blasticidin. Copy numbers of the *MycL* and *Dhfr* genes in the IR/MAR-negative transfectant were 3.1 and 4.1 per cell, respectively. *MycL*, *Dhfr*, and *IR* sequences in the IR/MAR-positive transfectant occurred at 84, 91, and 76 copies per cell, respectively, demonstrating balanced gene amplification from three parts of the transfected plasmid. Parallel with gene amplification, antibody protein expression increased five- to six-fold (Figure 5C; compare CN49-4 and CN49-12) in either the presence or absence of butyrate, which inhibits histone deacetylase and reverses epigenetic gene silencing. These results provide further evidence of the effectiveness of IR/MAR gene amplification.

When α(+) or α(−) plasmid set transfectants were selected in nucleotide-deficient medium with or without blasticidin, followed by culture in increasing concentrations of Mtx, a long HSR was generated at low-dose Mtx conditions inα(+) plasmid set cultures (Figure 5A; CN49-1 and 2). By contrast, amplification proceeded slowly (CN49-9), or cells were unable to adapt to the higher Mtx concentration (CN49-10), in the α(−) plasmid set cultures. Consistent with the gene amplification data, the antibody titer increased very little in the culture from the α(−) plasmid set (CN49-9 and CN49-10), but increased rapidly in the culture from the α(+) plasmid set (CN49-1 and CN49-2). In addition, the culture from the α(+) set grew and adapted to Mtx more rapidly than the culture from the α(−) set. Overall, compared with the conventional *Dhfr*/Mtx method, the IR/MAR-*Dhfr* fusion method resulted in more rapid gene amplification and production of larger amounts of antibody.

Real-time PCR results suggested that amplification of the three parts of the α(+) plasmid set was unbalanced in cells in the presence of higher doses of Mtx; the antibody gene (*MycL*) was not amplified in CN49-1 and CN49-2 cultures grown in 500 nM Mtx, while *Dhfr* and IR/MAR were highly amplified (Figure 5B). This tendency was consistently observed in two additional independent experiments (Figure S1); however, in these cases, unbalanced amplification was far more evident with the conventional *Dhfr*/Mtx method (CN59-14 and CN61-8) than with the IR/MAR-fusion method (CN59-13 and CN61-7), even at lower Mtx concentrations. With the *Dhfr*/Mtx method (CN49-9 and CN49-10), the PCR results (Figure 5B) suggested only a low level of gene amplification, whereas FISH analysis suggested a higher level (Figure 5A). In this case, the tiny FISH signals were sparsely scattered along the chromosome arm (Figure 4I). We hypothesized two explanations for the observed unbalanced gene amplification. The first hypothesis involves intra-allelic recombination inside the amplified plasmid repeat, which might remove the sequence that is unnecessary for cell growth. Such recombination would be activated by high-dose Mtx, which inhibits dihydrofolate reductase and causes folate deficiency followed by DNA damage. A cell with multiple long HSRs that were similar in appearance (Figure 4G), and a cell with multiple ring chromosomes composed of HSRs (Figure 4H), were observed in CN49-1 cultures grown in 500 nM Mtx. Generation of the former and latter structures may be explained by the mitotic checkpoint abrogation and enhanced recombination inside the HSR, respectively. Our second hypothesis was that unbalanced gene amplification might result from co-transfection of two plasmids, consisting of *MycL* located on one plasmid, and *Dhfr* and IR/MAR on the other (Figure 2A, B, and D). Co-transfection may result in generation of a predominant population of cells that bear amplified Dhfr and IR/MAR sequences only. We previously showed that any DNA can be co-amplified by co-transfection with the IR/MAR plasmid, and that the co-transfected DNA is ligated to the IR/MAR plasmid shortly after transfection [14]. As a result, the ratio of these two molecules may vary significantly. As discussed in the following sections, the former explanation was shown to be the case.

### Amplification and Expression of Antibody Using Plasmid Set ß

**Figure 6:**
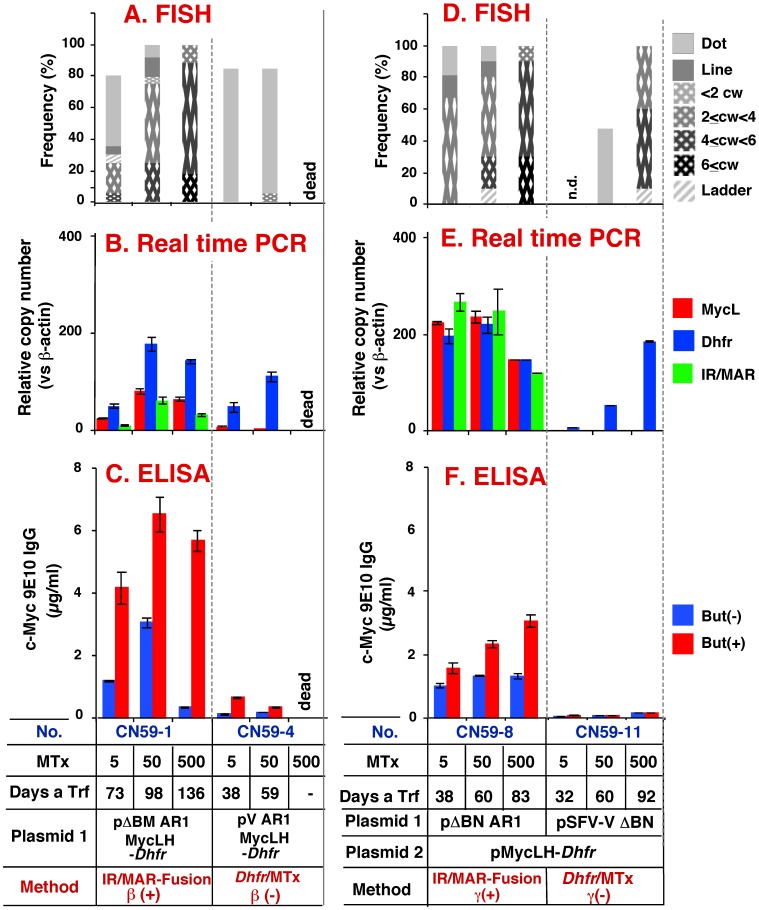
Amplification and antibody expression using plasmid sets ß and γ. CHO DG44 cells were transfected with plasmids 1 and 2 (as indicated) of sets β (A to C) and γ (D to F), and then selected by culture in the presence of 10 µg/ml BS, the indicated concentrations of Mtx, and the absence of G418. The transfectant number (No.), Mtx concentration (nM), and the amplification method, are indicated at the bottom of the figure. Cells reached confluence at the indicated number of days after transfection (Days a Trf). Cytogenetic structures were analyzed by FISH (A) (cw: chromowome width). Antibody expression was quantified by real-time PCR (B), and ELISA (C). Cells prepared for ELISA were grown in the presence (+) or absence (−) of 10 mM sodium butyrate (But). Error bars represent mean +/− S.D.

When plasmid set β was used, in which all the sequences are located on a single plasmid (Figure 2), the IR/MAR-containing ß (+) plasmid was amplified rapidly under low-dose Mtx selection (Figure 6A, CN59-1). By contrast, the ß (−) plasmid, which contains MAR but not IR, was amplified much later at a higher concentration of Mtx (Figure 6A, CN59-4) and extremely unbalanced gene amplification was observed, in which only *Dhfr* was amplified and *MycL* remained unamplified even at the lowest dose of Mtx (Figure 6B). Observation of such unbalanced amplification was surprising because all of the sequences were located in a single plasmid. This result strongly suggests that recombination between the plasmid sequences eliminated the antibody sequence in the IR(−) plasmid, which implies a further shortcoming of the conventional *Dhfr*/Mtx method. The amplification imbalance was much less pronounced in the transfectant from the IR/MAR ß (+) plasmid (CN59-1), which expressed a higher level of antibody even at the earliest stage of Mtx selection (Figure 6C).

### Amplification and Expression of Antibody Using Plasmid Set γ

Unlike plasmid set α, plasmid set γ has the *Dhfr* gene on the same plasmid as the antibody genes; therefore, strong Mtx selection pressure may be applied to the antibody-bearing plasmid, precluding unbalanced amplification even after co-transfection. The IR/MAR-positive plasmid set (γ(+)) was amplified rapidly under low-dose Mtx conditions (Figure 6D; CN59-8) and the amplification of each plasmid segment was balanced (Figure 6E). Conversely, the IR/MAR-negative (γ(−)) plasmid set was amplified later under high-dose Mtx conditions (Figure 6D, E; CN59-11), and the *Dhfr* gene was amplified while the *MycL* gene remained unamplified. Similar to the results for plasmid set ß(−), the unbalanced amplification observed for plasmid set γ(−) was surprising because *Dhfr* and *MycL* were ligated in a single plasmid at the time of transfection. These observations suggest that recombination between the amplified plasmid sequences, rather than plasmid co-transfection, is the primary cause of unbalanced gene amplification. Our results suggest that this undesirable recombination is infrequent if the IR/MAR-bearing plasmid is used, thus underscoring the merits of using the IR/MAR sequence during Mtx selection.

### Amplification and Expression of Antibody Using Plasmid Set δ

**Figure 7:**
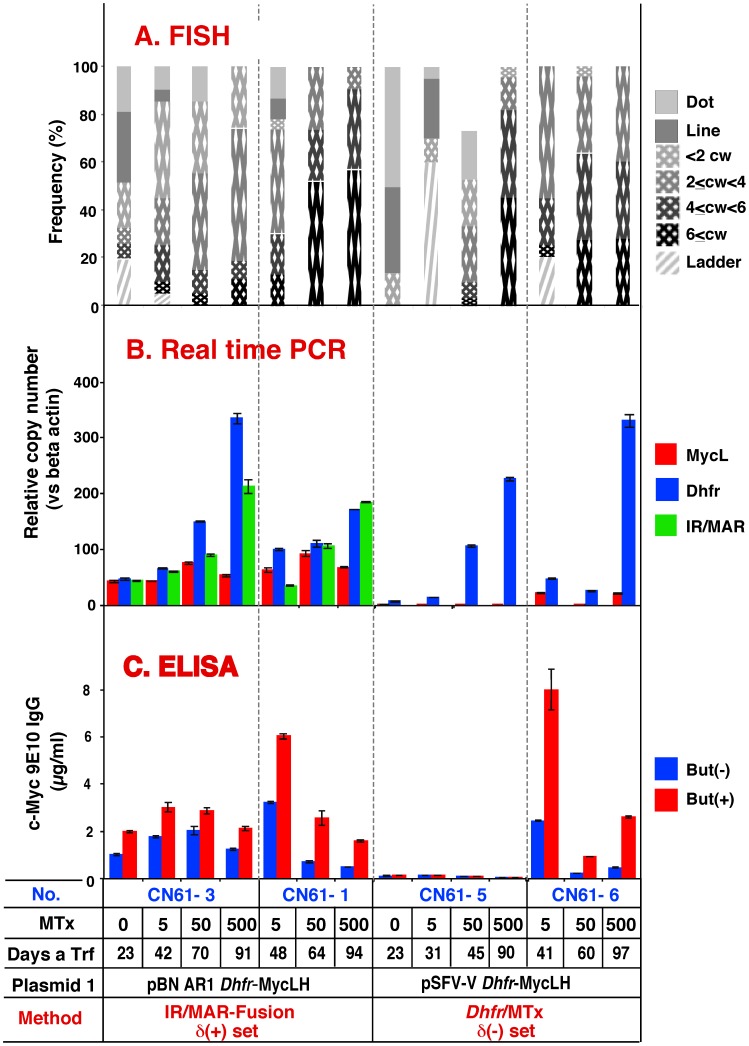
Amplification and antibody expression by using plasmid set δ. Plasmids 1 (indicated) of plasmid set δ were co-transfected and selected by culture in the presence of 10 µg/ml blasticidin, the indicated concentrations of Mtx, and the absence of G418. The transfectant number (No.), Mtx concentration (nM), and the amplification method are indicated at the bottom of the figure. Transfectants CN61-3 and -1, and CN61-5 and -6, differ by whether the selection was started from 0 or 5 nM Mtx in the nucleotide-deficient medium. Cells reached confluence at the indicated number of days after transfection (Days a Trf). Cytogenetic structures were analyzed by FISH (A) (cw: chromowome width). Antibody expression was quantified by real-time PCR (B), and ELISA (C). Cells prepared for ELISA were grown in the presence (+) or absence (−) of 10 mM sodium butyrate (But). Error bars represent mean +/− S.D.

Like plasmid set ß, (Figure 2J and K), all of the sequences in plasmid set δ are located within a single plasmid; however, the arrangement of the genes is different between the two sets. In plasmid set δ, the *Dhfr* gene is located close to IR, whereas the *MycH* and *MycL* genes are located further away from IR/MAR. In general, the results of amplification using plasmid set δ (Figure 7) were similar to those of the previous experiments and provided further evidence that inclusion of the IR/MAR sequence results in rapid and balanced amplification. However, the results obtained for plasmid set δ transfectant CN61-6 were inconsistent with the previous results. In this transfectant, the plasmid was amplified rapidly in the absence of IR/MAR and relatively large amounts of antibody were produced after low-dose Mtx (5 nM) selection. After induction by high-dose Mtx (50 nM and 500 nM), the culture was dominated by the cells that had unbalanced amplification; accordingly, antibody expression was dramatically decreased. The clones obtained from these cultures were then analyzed further.

### Acquisition and Stability of a High-Producer Clone

**Figure 8:**
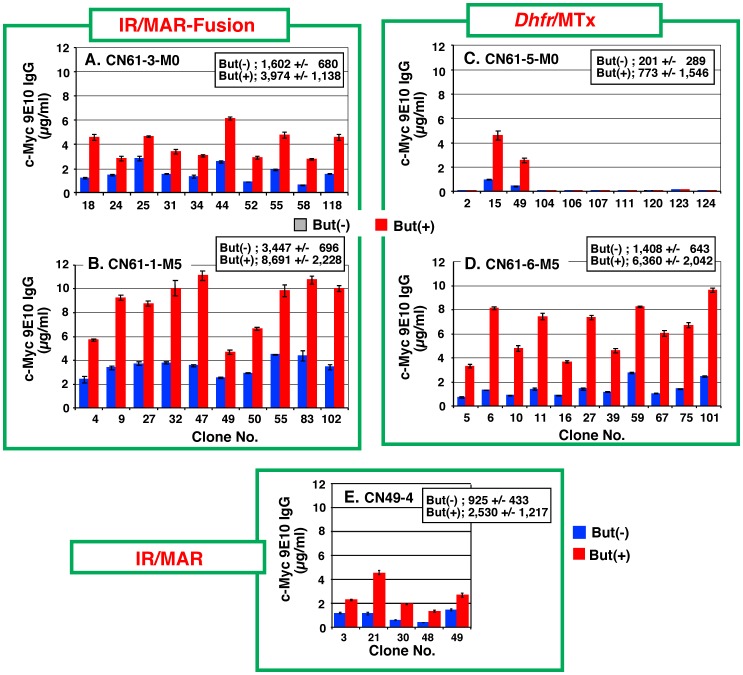
Clones obtained from plasmid set δ. Polyclonal transfectants (CN61-1, CN61-3, CN61-5, and CN61-6) that had been adapted to 0 nM (M0) or 5 nM (M5) Mtx in nucleotide-deficient medium (Figure 7), and polyclonal transfectant CN49-4 (Figure 5), were subjected to limiting dilution in 96-well plates. Wells containing a single colony were recorded and the culture medium from 100 (CN61-1, CN61-3, CN61-5, and CN61-6) or 50 (CN49-4) colonies for each transfectant was analyzed by ELISA (1st screening; data not shown). The ten (CN61-1, CN61-3, CN61-5, and CN61-6) or five (CN49-4) highest antibody-producing clones of each transfectant were selected; these cells were cultured in 96-well plates for 3 days in the presence or absence of 10 mM butyrate and the culture medium was analyzed by ELISA. The mean +/− S.D. are indicated in the upper right corner of each panel. Error bars represent mean +/− S.D.

Two polyclonal cultures from each of the IR/MAR-*Dhfr* fusion and *Dhfr*/Mtx methods were selected from the plasmid set δ transfectants (Figure 7), and 100 clones were first screened for each culture. Consequently, the ten highest producing clones for each culture were identified and antibody expression was measured in the presence or absence of butyrate (Figure 8A to D). In the absence of Mtx selection before the cloning procedure, the IR/MAR-*Dhfr* fusion method clones showed higher antibody expression (Figure 8A) than the *Dhfr*/Mtx method clones (Figure 8C). In addition, when low-dose Mtx (5 nM) selection was used before the cloning procedure, the IR/MAR-*Dhfr* fusion method clones showed the highest observed level of antibody expression (Figure 8B), while the *Dhfr*/Mtx method clones showed a moderate level of expression (Figure 8D) that was similar to or lower than the polyclonal culture (Figure 7). These data correlate with the lower stability of the *Dhfr*/Mtx method clones, and suggest a decrease in expression during the cloning procedure. Antibody expression in clones generated using the IR/MAR method (Figure 8E) was lower than that observed in the IR/MAR-*Dhfr* fusion method clones (Figure 8B), but higher than that obtained with the *Dhfr*/Mtx method (Figure 8C). These cloning results are in agreement with the distribution of cells expressing GFP, which was analyzed by flow cytometry (Figure 3).

**Figure 9:**
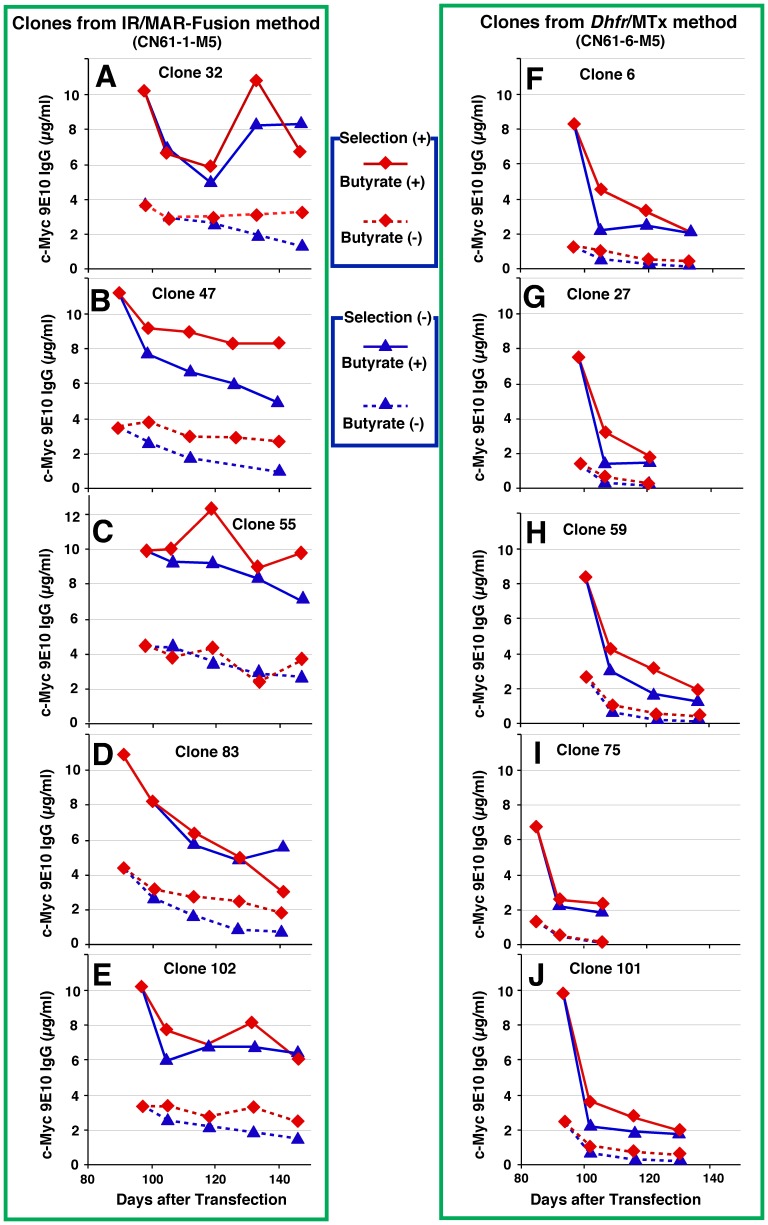
Stability of clones obtained using plasmid set δ. The five highest antibody-producing plasmid set δ clones generated using the IR/MAR-fusion (A to E) and *Dhfr*/Mtx (F to J) methods were selected and cultured in the presence (red lines) or absence (blue lines) of selective pressure (10 µg/ml blasticidin and 5 nM Mtx in the absence of nucleotides). After the indicated number of days post-transfection, cells were cultured in 96-well plates for 3 days in the presence (solid lines) or absence (dashed lines) of 10 mM butyrate and the culture medium was analyzed by ELISA.

Stability of the five highest antibody-producing plasmid set δ clones generated using the IR/MAR-fusion and *Dhfr*/Mtx methods during long-term culture was assessed (Figure 9). All clones obtained from the *Dhfr*/Mtx method rapidly lost antibody expression irrespective of the presence or absence of selective pressure (Figure 9F to J); the degree of instability was similar to previous reports [9], [11], [12]. By contrast, the clones obtained from the IR/MAR-fusion method were strikingly different. Although some clones exhibited moderate instability (Figure 9D), most were stable for 3 to 5 months after transfection, and their expression level was maintained above 50% of the initial level, even in the absence of selective pressure (Figure 9A to C, E).

### Implications of this Study

This study demonstrates that combination of the IR/MAR gene amplification method and the conventional *Dhfr/*Mtx method results in rapid plasmid amplification and generation of stable transfectants that produce large amounts of antibody. Our results suggest that a polyclonal population of transfectants growing in the presence of 5 nM Mtx may be obtained within 48 days (Figure 7; CN61-1); the clonal high-producer cell lines may be obtained within 90 days after the initial transfection (Figure 8) and may be stable for 150 days (Figure 9). One of the major drawbacks of the conventional *Dhfr*/Mtx method is the instability of the producer cells; we showed that this instability is primarily the result of enhanced recombination induced by high doses of Mtx, which leads to unbalanced amplification of different segments of the plasmid. Mtx is an inhibitor of DHFR that leads to folate deficiency followed by nucleotide-deficiency, which in turn may cause DNA strand breaks [25] and/or oxidative DNA damage [26]. Such an effect may plausibly explain the high level of recombination observed when high-dose Mtx treatment was employed. In fact, it was previously reported that Mtx induces high level karyotype instability among the cloned cell population [27]. Recombination appeared to occur more frequently at the amplified array than at other chromosomal sites, probably because the plasmid sequences were present as multiple copies, which allows intra-allelic homologous recombination. Consequently, a ring chromosome consisting solely of the plasmid HSR (Figure 4H) sometimes appeared.

The IR/MAR-fusion method generated more stable transfectants than the *Dhfr/*Mtx methods alone. The IR/MAR-bearing plasmid initially underwent spontaneous, extrachromosomal amplification in the transfected cells, followed by integration into the chromosome arm; this process did not require clastogenic Mtx treatment. The effect of the IR/MAR sequence on this initial amplification is highlighted by the observed difference between the CN49-4 and CN49-12 transfectants (Figure 5). Interestingly, Mtx treatment resulted in elongation of the amplified array and an increase in plasmid copy number (Figure 5, CN49-1, 2; Figure 6, CN59-1, 8; Figure 7, CN61-1, 3), suggesting that Mtx-induced the BFB cycle that was functioning only at a low level in CHO cells. Protein expression increased approximately in proportion to the increase in gene copy number. Although higher doses of Mtx also destabilized the structure generated by the IR/MAR-fusion method (Figure 5B, 6B, and 7B), the presence of IR/MAR significantly alleviated the destabilization. The most plausible explanation for this effect is the molecular structure of the amplified genes; specifically, the IR/MAR-fusion method spontaneously generates a tandem plasmid array before exposure to Mtx. By contrast, the *Dhfr*/Mtx method initially generates a low-copy number plasmid array on the chromosome arm, which is then amplified by the Mtx-induced BFB cycle. Therefore, the former structure appears to be more stable than the latter structure in the presence of Mtx. Identification of the molecular mechanism underlying this phenomenon will be an important subject for future study.

It was previously reported that both the MAR sequence [28]–[31] and the IR sequence [32] enhance expression from *cis*-linked genes. Our results showed that antibody expression from the ß(−) plasmid set, which contained only MAR (CN59-4; Figure?>;6), was lower than that from the β(+) set, which contained both IR and MAR (CN59-1). Amplification proceeded extensively in the ß(+) plasmid set, suggesting that gene amplification had a greater impact on the level of gene expression than the presence of MAR. Recently, several genomic sequences were successfully used to increase productivity of CHO cell transfectants. These methods include the MAR sequence, as well as the anti-repressor STAR element [33], transcription regulatory sequences from the Chinese Hamster EF-1alpha gene (named as a CHEF1 vector [34]), and the PiggyBac Transposon sequence [35]. Although these sequences were shown to stably increase productivity, they did not increase the transgene copy number. By contrast, our method, using a combination of IR and MAR sequences, did result in the stable amplification of the gene of interest.

The data presented here suggest that, compared with either the IR/MAR or conventional *Dhfr*/Mtx methods, the novel IR/MAR-*Dhfr* fusion method produces stable clones that express large amounts of recombinant protein with greater efficiency, speed, and ease at least in our experimental system. While this study was performed using adherent CHO cells in serum-containing medium, most industrial production of recombinant pharmaceuticals currently employs high-density suspension culture in serum-free medium. Usually, volumetric productivity is much higher under the latter condition; therefore, increasing productivity of the IR/MAR-*Dhfr* fusion method by modifying culture conditions and/or the culture medium should be investigated. In summary, the IR/MAR-*Dhfr* fusion method of gene amplification is an innovative tool for recombinant protein production.

## Supporting Information

Figure S1 **Amplification and antibody expression using plasmid set** α**.** CHO DG44 cells were co-transfected with pMycLH (plasmid 2) and pBNΔ AR1-*Dhfr* or pSFV-V-*Dhfr* (plasmid); cells were then selected by culture in the presence of 500 µg/ml G418 and the indicated concentrations of blasticidin (BS) and Mtx. The transfectant number (No.), Mtx concentration (nM), BS concentration (µg/ml), G418 concentration (µg/ml), and the method used, are indicated at the bottom of the figure. IR/MAR: IR/MAR method; Conv: conventional expression plasmid. Cells reached confluence at the indicated number of days after transfection (Days a Trf). Cytogenetic structures were analyzed by FISH (A) (cw: chromosome width). Antibody expression was quantified by real-time PCR (B), and ELISA (C). Cells prepared for ELISA were grown in the presence (+) or absence (−) of 10 mM sodium butyrate (But).
(TIF)
