# Supplementary figures and images for: Fusion of the Dhfr/Mtx and IR/MAR Gene Amplification Methods Produces a Rapid and Efficient Method for Stable Recombinant Protein Production
Source: PLoS One. 2012 Dec 31;7(12):e52990. doi: 10.1371/journal.pone.0052990 (PMC3534112; doi:10.1371/journal.pone.0052990)

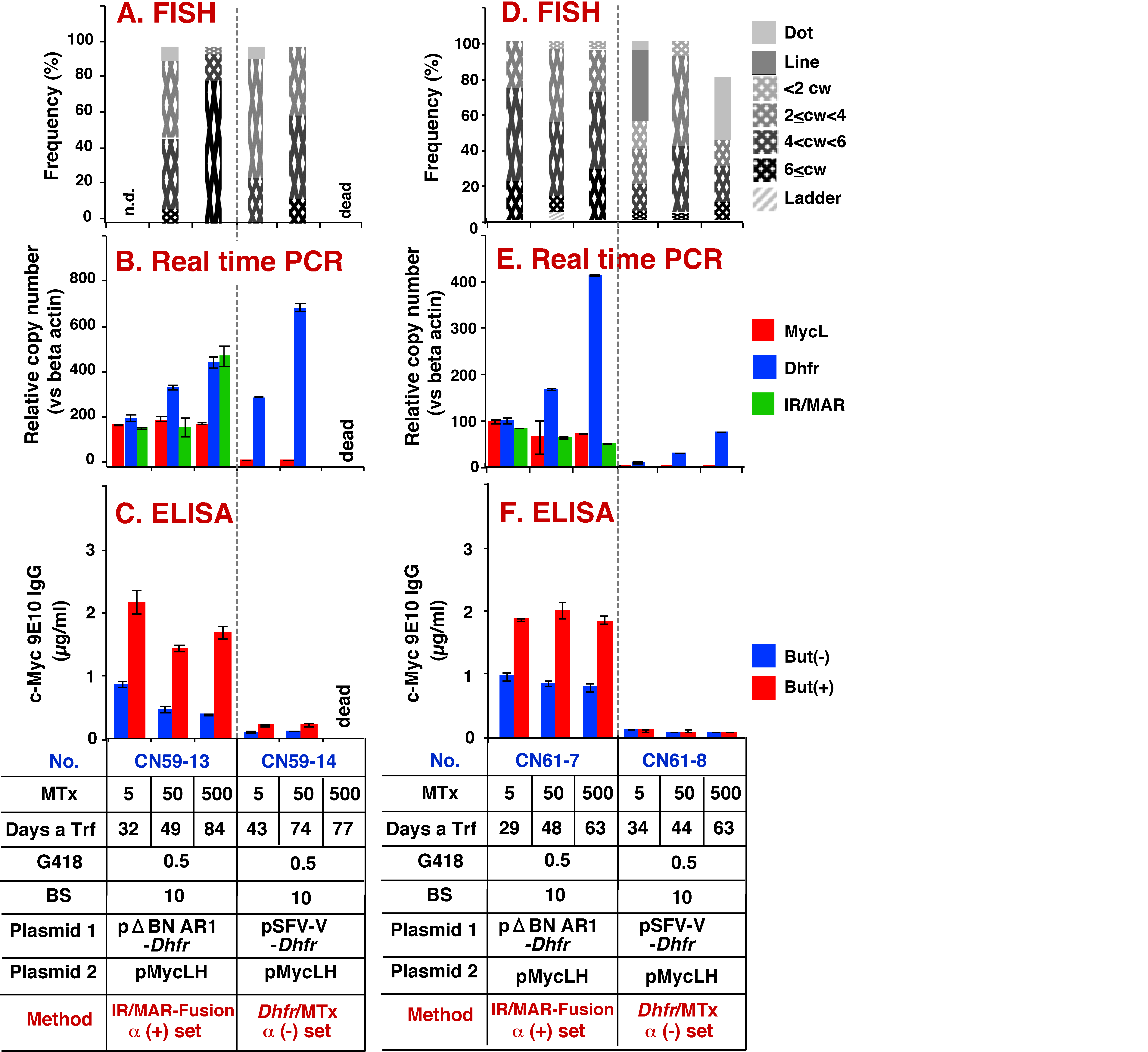

Supplement: Figure S1 — Amplification and antibody expression using plasmid set α. CHO DG44 cells were co-transfected with pMycLH (plasmid 2) and pBNΔ AR1-Dhfr or pSFV-V-Dhfr (plasmid); cells were then selected by culture in the presence of 500 µg/ml G418 and the indicated concentrations of blasticidin (BS) and Mtx. The transfectant number (No.), Mtx concentration (nM), BS concentration (µg/ml), G418 concentration (µg/ml), and the method used, are indicated at the bottom of the figure. IR/MAR: IR/MAR method; Conv: conventional expression plasmid. Cells reached confluence at the indicated number of days after transfection (Days a Trf). Cytogenetic structures were analyzed by FISH (A) (cw: chromosome width). Antibody expression was quantified by real-time PCR (B), and ELISA (C). Cells prepared for ELISA were grown in the presence (+) or absence (−) of 10 mM sodium butyrate (But). (TIF) [file pone.0052990.s001.tif]
